# Raising Well at Home: a pre-post feasibility study of a lifestyle intervention for caregivers and their child with obesity

**DOI:** 10.1186/s40814-020-00692-0

**Published:** 2020-10-06

**Authors:** Debra Haire-Joshu, Cindy Schwarz, Rebekah Jacob, Pat Kristen, Shelly Johnston, Karyn Quinn, Rachel Tabak

**Affiliations:** 1 Center for Obesity Prevention and Policy Research, the Brown School, Washington University in St. Louis, One Brookings Drive, CB 1196, Saint Louis, MO 63130 USA; 2 Center for Diabetes Translation Research, the Brown School, Washington University in St. Louis, One Brookings Drive, CB 1196, Saint Louis, MO 63130 USA; 3 Prevention Research Center in St. Louis, the Brown School, Washington University in St. Louis, One Brookings Drive, CB 1196, Saint Louis, MO 63130 USA; 4 Centene Center for Health Transformation, the Brown School, Washington University in St. Louis, One Brookings Drive, CB 1196, Saint Louis, MO 63130 USA

**Keywords:** Childhood obesity, Caregiver, Home visiting, Peer coaches, Medicaid population

## Abstract

**Background:**

Few efficacious pediatric obesity interventions have been successfully translated and sustained in real-world practice, often due to inadequate fit with the priorities of under-resourced populations. Lifestyle interventions, which incorporate tailoring of essential weight loss ingredients and adaptation of mode and intensity to the living circumstances of children with obesity, are needed. The purpose of this pilot study was to test the feasibility and efficacy of a tailored lifestyle intervention for caregivers and their children with obesity, conducted in partnership with Envolve, Inc., a family of comprehensive health solutions and wholly owned subsidiary of Centene Corporation.

**Methods:**

This 6-month pilot study employed a pretest-posttest design to assess the impact of a tailored lifestyle intervention delivered by peer coaches on (a) caregiver and child weight impacted by changes in dietary intake, walking, and screen time; (b) changes in the home environment; and (c) caregiver engagement and satisfaction. The intervention was delivered via 3 core home visits every 4–6 weeks, with additional support via text.

**Results:**

The majority of caregivers were female (95.2%) and Black (73.7%). Children had median age of 11.1 years and majority were female (57.6%), with a median BMI near the 99th percentile (Mdn 98.8, IQR 3.5) or 118.3% (IQR 35.8) of the 95th percentile for their sex and age. Participants expressed high satisfaction with the program (mean range 96.7–100.0% agreement on satisfaction items). From baseline to post, caregivers’ BMI decreased by 1.8% (*p* = 0.016, *r* = 0.22), while children’s BMI percentile *z*-score decreased significantly (*p* = 0.023, *r* = 0.18) and BMI percent of the 95th percentile remained constant (*p* = 0.05, *r* = 0.15). Caregivers and children decreased sugar-sweetened beverage intake (*p* = 0.026, *r* = 0.22; *p* = 0.006, *r* = 0.23, respectively), reduced presence of soda in the home (*p* = 0.002, *g* = 0.43), and decreased screen time (*p* = 0.046, *g* = 0.22). Other eating and walking behaviors remained stable for caregivers and child.

**Conclusion:**

The Raising Well at Home pilot demonstrated that tailored lifestyle interventions, delivered by peer coaches in the home and via text, are feasible and can improve weight, eating, and environmental measures of caregivers and children with obesity. Future work should determine the effectiveness, sustainability, and scalability of this intervention in sites located across the country.

**Trial registration:**

ClinicalTrials.gov (NCT04224623). Registered 9 January 2020—retrospectively registered.

## Key messages regarding feasibility

- What uncertainties existed regarding the feasibility?
  Common findings of low participation rates among participants from under-resourced communities suggest more work is needed to reach caregivers of children with obesity, perhaps through channels in which they are already engaged, and to tailor lifestyle interventions with life priorities.
- What are the key feasibility findings?
  This pilot demonstrated a lifestyle change intervention, tailored to the context of Medicaid members and delivered by a peer coach through a minimal number of home visits, was met with high satisfaction by participants and yielded a significant reduction in caregiver weight and impacted child BMI *z*-score and BMI percent of the 95th percentile.
- What are the implications of the feasibility findings for the design of the main study?
  This pilot provides further evidence of the feasibility and impact of a tailored lifestyle intervention that can be delivered by peer coaches through Envolve, with the potential to reach caregivers and their children with obesity across the nation.

## Background

Few efficacious pediatric obesity interventions have been successfully translated and sustained in real-world practice [1]. This is significant since childhood obesity affects more than 18% of children in the USA, making it the most common chronic disease of childhood [1] and a contributing factor to children being diagnosed with co-morbid conditions such as diabetes [2, 3]. There is also a strong association between childhood and adult obesity; children with obesity are about 5 times more likely to have obesity as adults than healthy weight children, making early intervention critical [1, 4]. Additionally, obesity tends to be present across generations, with children with obesity more likely to have at least one parent who also has obesity [5]. Parent/caregiver-child interactions influence a child’s risk for obesity through multiple pathways, influenced not only by family factors but also by sociocultural, community/environmental, and socioeconomic status [6, 7]. Lifestyle interventions for children with obesity need to target both caregivers and children for change while addressing the context within which they live.

Intensive lifestyle interventions to treat children with obesity often require repeated visits to clinics or hospitals, which leads to treatment attrition and decreased efficacy [8, 9]. Challenges to treatment include cost, time, transportation, and interference with life activities, all of which are heightened among under-resourced families dealing with additional social needs such as shortages of food, unstable housing, and inability to pay for utilities [8, 9]. These competing and immediate priorities limit access to and uptake of intensive interventions, and contribute to attrition, further exacerbating obesity disparities. Selecting a resource-intensive intervention for use with families living in under-resourced communities reflects inadequate consideration of fit with real-world needs of the child served. This suggests the importance of tailoring of essential evidence-based ingredients needed for weight loss and adaptation of mode and intensity of delivery to match the living circumstances and priorities of the caregiver and their child with obesity.

One promising method to better align interventions with real-world needs and address disparities is to involve peer coaches in the adaptation and delivery of childhood obesity interventions [5, 10]. Peer coaches, also referred to as community health workers or lay health educators, are often defined as individuals who (1) participate in some capacity in health promotion, (2) receive training for intervention delivery but have no formal professional healthcare training, and (3) have an existing relationship or other connection with the population receiving care [11, 12]. They are also typically from, and understand, the community they serve [13, 14] and may be more easily integrated into systems of care. Peer coaches are often trained to offer emotional (e.g., active listening), affirmational (i.e., validating and supporting self-efficacy), and practical/informational (i.e., “lived experience”) support to families [15]. They have been effectively used to deliver numerous health-related interventions addressing a variety of health conditions in home, clinic, school, and community settings [13, 14, 16]. Studies have suggested obesity interventions delivered by peer coaches may be effective for preventing and treating adult and childhood obesity, especially in under-resourced populations [17–20]. The delivery of lifestyle interventions by peer coaches further assures tailoring of content consistent with life priorities of the families.

To eliminate disparities in the treatment of childhood obesity in populations most in need, interventions also need be scalable to assure wide reach [21–24]. Partnering with organizations that offer peer coach approaches in reaching under-resourced families can enhance convenience, accessibility, and availability of lifestyle interventions for children and families with obesity, while providing a structure to disseminate successful interventions for broad reach [25]. Envolve, Inc. is a family of comprehensive health solutions serving millions of Medicaid patients across the USA. Envolve is a wholly owned subsidiary of Centene Corporation, a diversified, multi-national healthcare enterprise that provides a portfolio of services to government-sponsored and commercial healthcare programs, focusing on under-insured and uninsured individuals [26, 27]. Medicaid is a safety-net health insurance program in the USA serving adults, children, pregnant women, elderly adults who meet low-income criteria, and people with disabilities.

We partnered with Envolve to develop and pilot the Raising Well at Home Program for children with overweight or obesity and their caregivers. This program trained peer coaches to tailor a lifestyle intervention for both the caregiver and child delivered through three core home visits and check-in contacts via texting and video chat over 6 months. This pilot was developed as an alternative to the Raising Well telephone health coaching program offered through Envolve, which uses motivational interviewing delivered by healthcare professionals (e.g., dietitians) to promote lifestyle change for caregivers and their child with obesity [28, 29]. The Raising Well at Home pilot study assessed the efficacy and feasibility of a peer coaching lifestyle intervention on (a) caregiver and child weight impacted by changes in dietary intake, walking, and sedentary behaviors associated with screen time; (b) changes in the home environment to support health behaviors; and (c) caregiver engagement and satisfaction.

## Methods

### Raising Well at Home

The Raising Well at Home pilot study was initiated by Envolve in 2015 and trained peer coaches to deliver a lifestyle intervention through home visits, tailored to the needs of caregivers and their children (ages 2–17) with overweight or obesity. The evidence-based intervention was adapted from prior work [30–32] to match caregiver and child needs and designed to enhance the relevance to the family context. During collaborative meetings, staff from the Raising Well parent program and the research team worked to identify essential lifestyle content to assure the intervention was consistent with the needs of the families and fit within the structure of the Envolve programs, optimizing opportunity to embed the intervention within standard practice. The Raising Well at Home intervention was also designed to be consistent with the parent Raising Well program, and available as another option to meet the diverse priorities of the Medicaid families served.

#### Theoretical approach and content

Theoretically based interventions that focus on small, consistent changes in obesity-related eating and activity behaviors, with ongoing support, have been shown to be effective in reducing weight [30, 31, 33]. The theoretical basis for Raising Well at Home was Social Cognitive Theory, emphasizing (1) intrapersonal influences (e.g., constructs of self-assessment, reinforcement, and behavioral capability), (2) interpersonal influences (e.g., observational learning/caregiver model for child), and (3) how these interactions influence, or are influenced by, the environment of the caregiver (e.g., home environment) [34–36]. Given the competing priorities of this population, a limited number of select eating and activity behaviors were identified for the intervention. These essential behaviors focused on reducing sugary beverage intake, promoting appropriate portion sizes, improving fruit and vegetable intake, increasing walking, and reducing screen time/sedentary activity.

#### Intervention structure and dose

The Raising Well at Home intervention was delivered via 3 core home visits designed to be delivered every 4–6 weeks, with additional support via text or video chat over 6 months, beginning after completion of the baseline assessment. Importantly, the peer coach had flexibility in delivering and tailoring the lifestyle intervention to family circumstances and priorities as appropriate. The home visits were designed to engage the whole family, build skills, and promote changes in the home environment to support lifestyle behavior change. The peer coach and the caregiver/child dyads were encouraged to work together to achieve goals sensitive to real-world context. Table 1 provides additional intervention details.

**Table 1:** Raising Well At Home intervention

Name	Raising Well-Health at Home
Rationale	Interventions that meet families where they are, are offered over time in a variety of ways, and recognize families’ competing priorities can better serve families with Medicaid
Provider	Peer coaches
Mode	Home visits (*N* = 3): scheduled to meet the needs of the family Behavioral check ins: by phone or text, frequency determined by peer coach and/or participant
Intervention design	Home visits based on social cognitive behavior change theory including the following: (a) observation of the parent, child, and/or home environment to assure *relevance* (e.g., availability of healthy food at home or in community); (b) *participatory* parent-child activities to reinforce behavior change (e.g., parental modeling of positive eating patterns); (c) *pragmatic* changes for a supportive physical home environment (e.g., remove TV from bedrooms); (d) *feedback* on progress through self-assessment (e.g., goal setting); and (e) *reinforcement* of family strengths
Home visit content	Visit 1—reducing sugar-sweetened beverage intake, and beverage access: self-assessment of beverage intake; measure sugar in high calorie beverages; compare sugar and calories in different beverages; discuss and identify healthier drink options; set goals and identify strategies; improve beverage environment Visit 2—improving portion control, healthy snacking, and food environment: read food labels to identify healthy portions; self-assess nutritional quality of foods; discuss and identify healthy portions; set goals and identify strategies for healthy portions and modifying home food environment to support healthy choices Visit 3—increasing walking; reduce screen time-sedentary behaviors: self-assess current walking patterns and screen time-sedentary behaviors; do activity with the family; set up activity tracker; discuss and identify how to be active; set goals and strategies for achieving
Tailoring	The structure of the visit is flexible so non-health-related social needs can be prioritized. Since the visit is taking place in the participant’s home, and the peer coach is familiar with the community, the content is tailored to build on the family’s home and neighborhood environment

### Peer coach recruitment and training

Raising Well at Home peer coaches understood the challenges faced by the under-resourced communities in which they resided and were trained to provide ongoing practical, social, and emotional support, and to communicate health information in a culturally meaningful, understandable way to caregivers and children. They were recruited from communities in three states served by the parent Raising Well program (*N* = 12; five in Florida; five in Louisiana; two in Missouri). Raising Well at Home training by research staff included the following: (1) intervention background and rationale, (2) evidence base for successful pediatric and family weight loss interventions, (3) the importance of caregiver lifestyle and influence on child weight and development, (4) overview of Raising Well at Home protocol, and feedback from trainers. Trainings took place via video conference or in person over 18 h, consistent with training sessions often offered through Envolve. Follow-up booster trainings were provided as needed.

### Study design and setting

This pilot study employed a pretest-posttest design [37] to assess the feasibility and efficacy of the Raising Well at Home pilot intervention delivered by peer coaches on weight (caregiver/child), behaviors (eating, walking), and the home environment.

### Child-caregiver recruitment

This study was implemented in partnership with the parent Raising Well programs located in three states: Florida, Louisiana, and Missouri. For inclusion, caregivers had to be at least 18 years old, live in one of the states offering the Raising Well program, and be the primary caregiver to at least one child 2–17 years old served by an Envolve Medicaid Health Plan with International Classification of Diseases, Ninth Revision (ICD-9) coding for overweight or obesity. Caregivers had to be able to give informed consent. If more than one child within a household was eligible to participate, all children were invited to participate. Exclusion criteria for both caregivers and children included inability to speak English or Spanish.

The Raising Well at Home pilot was offered in zip codes that were geographically accessible to a peer coach; Envolve representatives identified the names of eligible participants in these target zip codes through claims data. The research team mailed potential participants information about the study which was followed by a recruitment call by the peer coach. At least three call attempts were made to reach each potential participant. When a caregiver was reached by phone, the peer coach described the study and obtained verbal consent. If the caregiver consented to a study visit, the peer coach scheduled the visit for a time convenient for the family. Participant recruitment occurred between July 2016 and March 2019. The intervention concluded and data analysis was completed in 2019. Participants received a $25 gift card for completing data collection at each of two time points (i.e., baseline and post-intervention). This study was approved by the Washington University in St. Louis Institutional Review Board, by the Louisiana Department of Health Institutional Review Board, and by participating Medicaid health plans and state agencies responsible for the review and approval of member material.

### Data collection

All measures were completed in the home setting and required 30–40 min. Signed, written consent was completed at the baseline visit; children of age provided assent. Measures were completed on a paper survey with the same caregiver pre- and post-intervention delivery. The written questions were completed by the caregiver, while the measures of behaviors, such as diet and activity, were asked verbally so the child could assist in answering the questions, if able. Surveys were scanned and sent to the research team to enter into a secure web application for building and managing surveys and databases. The average time between the pretest and the posttest survey was 188.3 days (or approximately 6 months) (range = 121 to 383 days).

### Measures

#### Fidelity, implementation, and satisfaction measures

To ensure that each protocol was appropriately delivered, peer coaches completed lesson plan checklists documenting delivery of content and perceived engagement with the caregiver. Information from the checklist included visit topic and rationale for choice of that topic, completion of session objectives, time allotted, and quality of visit. Additionally, peer coaches were encouraged to address compatibility with caregiver-child needs, level of complexity, difficulty or expertise required to deliver the content, adequacy of training to conduct the home visit, and satisfaction with visit.

To assess program delivery by the peer coach, intervention contacts were audio-recorded and reviewed by research staff to assess content. Peer coaches also recorded all call and home visit attempts along with details related to participant recruitment, retention, and engagement with the intervention. The peer coach participated in monthly consultations with research staff regarding intervention delivery.

The survey administered at the post-intervention time point asked caregivers to rate satisfaction with their peer coach and with Raising Well at Home on a 5-point Likert scale (1 = very satisfied/5 = very dissatisfied). Caregivers were also asked if they met in person, texted, and/or video chatted with their peer coach, and asked to rate that experience on a 5-point Likert scale (strongly agree/strongly disagree).

#### Sociodemographic measures

Caregivers reported sociodemographic measures including age, marital status, number of children, and education.

### Weight and behavioral outcomes

The caregiver and the child’s height and weight were measured by trained staff in the home with the same scale and stadiometer at the pre- and post-intervention time points. Staff followed the procedures in accordance with Centers for Disease Control and Prevention recommendations [38, 39].

Caregiver and child beverage intake was determined using a modified Brief Questionnaire to Assess Habitual Beverage Intake (BEVQ-15) [40–44]. The measure asked about how often and how much 15 different beverages were consumed. Two questions were also asked to assess fruit and vegetable intake.

We asked two questions to assess bouts of walking during the past week: “How many times did you walk for at least 10 min for fun, relaxation, exercise, or to walk the dog?” [45] and “How many times did you walk to get some place that took you at least 10 min?” [46].

### Home environment

The Activity and Diet scale of the Home Self-administered Tool for Environmental survey (HomeSTEAD) and measures from the Healthy Eating and Active Living Taught at Home study were used to assess modifications of the home environment to support healthy eating and activity [47–49]. This included availability and accessibility of sweet and salty snacks and of soda (e.g., I have soda in the home that is easy to get to and in plain sight, easy to get to but out of sight, hidden and out of reach, or no soda in the home).

### Statistical analyses

We calculated child BMI percentiles (and *z*-scores) based on CDC 2000 growth charts (ages 0 to < 20 years) for sex and age. As recommended to detect change in samples with very high child BMI percentiles [50, 51], the percent of the 95th BMI percentile was calculated to represent BMI percentile relative to the 95th percentile. Each child’s BMI is divided by the BMI at the 95th percentile for the child’s sex and age and multiplied by 100. For example, a 10-year-old male with a BMI of 28 kg/m^2^ would be 127% of the 95th percentile for his sex and age (28/22 × 100). As such, this measure is much more sensitive to change among children in the highest BMI percentiles.

BEVQ data were processed in the following order to calculate the average ounces per day of sugar-sweetened beverages (SSBs) consumed by participants. Categories of frequency were converted into continuous “average times per day” (e.g., 1 time per week category = 1/7 or 0.143 average times per day). Categories of ounces at each time of consumption were converted to continuous variables to represent “ounces” (e.g., category “16 fl oz (2 cups)” = 16.0). Average times per day for each beverage was then multiplied by ounces to represent “average ounces per day” consumed. To quantify average daily ounces of SSB consumed, beverage categories containing added sugars were summed (sweetened juice beverages and drinks, regular sugar-sweetened carbonated beverages, sweet tea, sweetened coffee, and energy drinks).

Descriptive statistics were conducted in order to understand the overall frequencies and distributions of demographic and outcome data among caregivers and children. Bivariate analyses were conducted to detect any changes in main outcomes from baseline to post-assessment. McNemar tests were used to compare matched proportions of nominal outcomes between baseline and post-assessment. Assumptions of normality for paired *t* tests were not met, and therefore, Wilcoxon paired signed-rank tests, robust to nonnormality issues, were used to measure mean rank changes in continuous variables between time points. Pre-post differences were examined in terms of effect size. For Wilcoxon signed-rank tests (continuous data), effect size was calculated as $r=\frac{z}{\sqrt{n(obs)}}$. Interpretations for *r* are 0.1 small, 0.3 medium, and 0.5 large effect [52]. For McNemar tests (proportional data), Cohen’s *g* for effect size was calculated as $g=\left(\frac{b}{b+c}\right)-0.5$ for a 2 × 2 table where *a* and *d* are the concordant cells and *b* and *c* are the discordant cells. Interpretations for *g* are 0.05 to 0.15 small, 0.15 to < 0.25 medium, and > 0.25 large [53].

All data cleaning, management, and analyses were completed using R (3.6.1) [54] in R Studio (version 1.2.1335) [55] using the *tidyverse* (1.2.1), *AGD* (0.39) [56], *rcompanion* (2.3.25) [57], and *childsds* packages (0.7.4) [58].

## Results

**Fig. 1:**
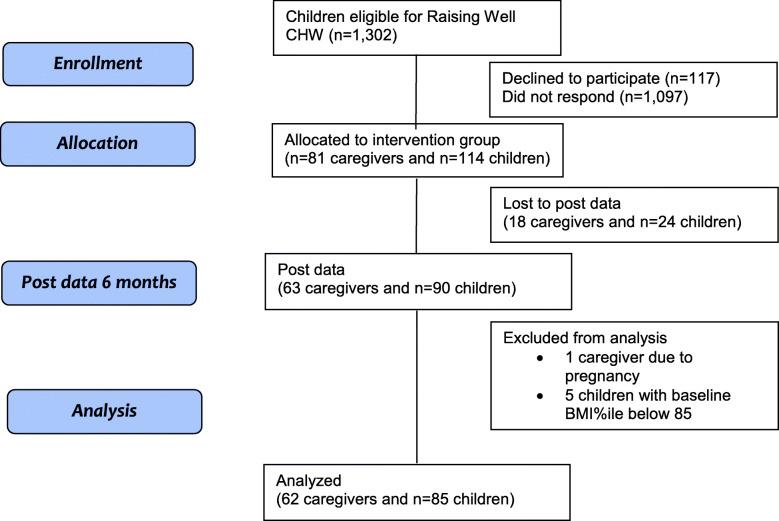
CONSORT diagram

We obtained *N* = 1302 eligible names from Envolve for children with overweight or obesity (aged 2 to 17 years) in Louisiana (*N* = 460), Missouri (*N* = 401), and Florida (*N* = 441). We were unable to contact 84.3% of identified names: 47.8% were unable to be contacted due to disconnected/wrong numbers (*n* = 622); messages were left with 36.5% (*n* = 475) who did not respond. Of the 205 names of caregivers reached, 44.4% (*n* = 91) declined and 55.6% (*n* = 114) enrolled. Among those enrolled, complete data was obtained on 77.7% (*n* = 63) of caregivers and 78.9% of children (*n* = 90) and 6 were excluded from analysis (1 caregiver due to pregnancy and 5 children with a BMI percentile below 85). Figure 1 shows the number of all enrolled, unable to contact, dropout, and complete data.

Almost all of caregiver participants were female (95.2%), and the majority were African American or Black (73.7%) (see Table 2). The median age for caregivers was 38.0 years (IQR 10.5). Most (67.9%) were single (or not currently married or living with a partner). Employment status varied among caregivers with 44.1% working 30 h or more per week, 20.3% working less than 30 h, and 35.6% not currently employed outside of the home. Most (82.3%) were receiving assistance from at least one federal government benefit programs (e.g., WIC, SNAP) in addition to Medicaid. The median BMI for caregivers was 35.5 (IQR 10.3) with most (83.6%) falling into the obese range (BMI 30.0 or above) (see Table 3).

**Table 2:** Caregiver and children demographics at baseline

Caregiver characteristic	***N*** = 62
Age in years: median (IQR)^a^; *N* = 59	38.0 (10.5)
Sex: *n* (%)	
Female	59 (95.2)
Male	3 (4.8)
Race: *n* (%)	
Black or African American	42 (73.7)
White	13 (22.8)
Multiple or other races	1 (1.8)
Prefer not answer	1 (1.8)
Hispanic ethnicity: *n* (%)	
Hispanic or Latino	7 (13.7)
Not Hispanic or Latino	39 (76.5)
Prefer not answer	5 (9.8)
Marital status: *n* (%)	
Married or living with a partner	18 (32.1)
Never married, divorced, separated, or widowed	38 (67.9)
Education (highest achieved): *n* (%)	
High school diploma or less	26 (44.8)
Some college, or technical/vocational school	20 (34.5)
College or university graduate, or higher	12 (20.7)
Working hours per week: *n* (%)	
Not employed outside of home	21 (35.6)
Employed 30 h per week or fewer	12 (20.3)
Employed more than 30 h per week	26 (44.1)
Family receives program assistance (WIC, SNAP, etc.)^b^: *n* (%)	51 (82.3)
Barriers that prevent caregiver from being able to manage child’s condition: *n* (%)	
No barriers	41 (66.1)
Financial constraint	10 (16.1)
Transportation	6 (9.7)
Food insecurity	5 (8.1)
Housing	4 (6.5)
Support	3 (4.8)
Religious/ethnic beliefs	1 (1.6)
Other barrier	2 (3.2)
**Child characteristics**	***N*** **= 85**
Age in years: median (IQR)^a^; *N* = 59	11.1 (4.1)
Sex: *n* (%)	
Female	49 (57.6)
Male	36 (42.4)
Race: *n* (%)	
Black or African American	55 (70.5)
White	19 (24.4)
Multiple or other races	2 (2.6)
Prefer not answer	2 (2.6)
Hispanic ethnicity: *n* (%)	
Hispanic or Latino	16 (21.9)
Not Hispanic or Latino	49 (67.1)
Prefer not answer	8 (11.0)
Caregiver worried that child is overweight: *n* (%)	
Agree a little/agree a lot	49 (65.3)
Neutral/disagree a little/disagree a lot	26 (34.7)

^a^*IQR* interquartile range

^b^In addition to Medicaid: *WIC* Special Supplemental Nutrition Program for Women, Infants, and Children, *SNAP* Supplemental Nutrition Assistance Program

**Table 3:** Baseline and post-assessment comparison of caregiver and child outcomes

	Baseline	Post	***p*** value^**a**^	Effect^**b**^
**Caregivers**	***N*** **= 62**	***N*** **= 62**		
BMI kg/m^2^: median (IQR); *N* = 61	35.5 (10.3)	34.9 (11.3)	0.016	0.22
Daily sugar-sweetened beverage intake (oz): median (IQR); *N* = 50	10.1 (27.1)	5.7 (14.6)	0.026	0.22
Times caregiver ate vegetable and fruit (combined), previous day: median (IQR); *N* = 56	2.0 (2.0)	2.0 (2.0)	0.674	0.04
Times caregiver walked to get some place that took at least 10 min, previous week			0.724	0.13
0–2 times per week	39 (84.8%)	48 (80.0%)		
3 or more times per week	7 (15.2%)	12 (20.0%)		
Times caregiver walked for at least 10 min for fun, relaxation, exercise, previous week			0.752	0.10
0–2 times per week	33 (70.2%)	44 (75.9%)		
3 or more times per week	14 (29.8%)	14 (24.1%)		
Hours a day caregiver watches TV or uses a computer/laptop/tablet/iPad (for playing games, emailing, chatting, or surfing on the net) in free time			0.332	0.15
Less than 2 h per day	24 (39.3%)	29 (47.5%)		
2+ h per day	37 (60.7%)	32 (52.5%)		
Times caregiver ate fast food for a main meal each week			0.179	0.20
Seldom/never	12 (20.0%)	14 (23.0%)		
1 time per week	16 (26.7%)	22 (36.1%)		
2+ times per week	32 (53.3%)	25 (41.0%)		
Soda in the home is			0.002	0.43
Easy to get to (in plain sight or out of sight)	37 (60.7%)	24 (40.0%)		
Not in home or hidden and out of reach	24 (39.3%)	36 (60.0%)		
Sweet/salty snacks in the home are			0.202	0.33
Easy to get to (in plain sight or out of sight)	58 (93.5%)	52 (86.7%)		
Not in home or hidden and out of reach	4 (6.5%)	8 (13.3%)		
**Children**	***N*** **= 85**	***N*** **= 85**		
BMI percentage of the 95th percentile (%): median (IQR); *N* = 83	118.3 (35.8)	121.9 (31.8)	0.050	0.15
BMI percentile: median (IQR); *N* = 83	98.8 (3.5)	98.7 (3.4)	0.010	0.20
BMI *z*-score: median (IQR); *N* = 83	2.3 (0.8)	2.2 (0.7)	0.023	0.18
Daily sugar-sweetened beverage intake (oz): median (IQR); *N* = 70	4.7 (17.9)	2.5 (10.6)	0.006	0.23
Times child ate vegetable and fruit (combined), previous day: median (IQR); *N* = 78	2.0 (2.0)	2.0 (1.0)	0.388	0.07
Times child walked to get some place that took at least 10 min, previous week			0.803	0.06
0–2 times per week	57 (85.1%)	66 (79.5%)		
3 or more times per week	10 (14.9%)	17 (20.5%)		
Times child walked for at least 10 min for fun, relaxation, exercise, previous week			0.480	0.11
0–2 times per week	50 (75.8%)	58 (73.4%)		
3 or more times per week	16 (24.2%)	21 (26.6%)		
Hours per day child watches TV or uses computer/laptop/tablet/iPad (for playing games, emailing, chatting, or surfing on the net) in free time			0.046	0.22
Less than 2 h per day	27 (32.9%)	40 (47.6%)		
2+ h per day	55 (67.1%)	44 (52.4%)		
Times child ate fast food for a main meal each week			0.007	0.31
Seldom/never	9 (11.4%)	19 (23.2%)		
1 time per week	27 (34.2%)	33 (40.2%)		
2+ times per week	43 (54.4%)	30 (36.6%)		

^a^*p* value from McNemar tests for paired nominal data and Wilcoxon signed-rank tests for paired continuous data

^b^For Wilcoxon signed-rank tests (continuous data), effect size was calculated as $r=\frac{z}{\sqrt{n(obs)}}$. Interpretations for *r* are 0.1 small, 0.3 medium, and 0.5 large effect. For McNemar tests (proportional data), Cohen’s *g* for effect size was calculated as $g=\left(\frac{b}{b+c}\right)-0.5$ for a 2 × 2 table where *a* and *d* are the concordant cells and *b* and *c* are the discordant cells. Interpretations for *g* are 0.05 to 0.15 small, 0.15 to < 0.25 medium, and > 0.25 large

Child participants had a median age of 11.1 years (IQR 4.1), and over half (57.6%) were female. Like caregivers, most were African American or Black (70.5%). Children’s median BMI was nearly the 99th percentile (Mdn 98.8, IQR 3.5) for their sex and age, or 118.3% (IQR 35.8) of the 95th percentile for their sex and age. A majority of caregivers (65.3%) agreed they were worried that their child (or each of their children separately) was overweight or had obesity.

### Intervention feasibility

Caregivers and children completed 2.57 home visits [SD = 0.84]; the average length of visits was 70 min. Almost all participants completed visits in person (98.1%) and also texted (83.0%) with their peer coach, while only 3.9% used video chat to connect with their peer coach. Caregivers agreed that texting with their peer coach (100%) and in-person visits (92.0%) were simple to do.

Caregivers expressed high satisfaction with the program and with their peer coach. Almost all (mean range 96.7–100%) were satisfied or very satisfied with their peer coach relevant to (1) the overall service received from the program, (2) experience and knowledge, (3) attention to detail, (4) personal manner (politeness, respect, sensitivity, friendliness), (5) concern for caregivers’ comfort, (6) support and understanding, (7) willingness to schedule calls at a time that worked for caregiver, and (8) helpfulness. In addition, 96.7% of caregivers would recommend the program to a family member. Caregivers reported convenient access to text messaging, smart phone, wifi, and/or a computer/tablet with wifi (between 85.4 and 93.8%). When asked about preferences for how to receive the program, in-person visits was the top-ranked preference (78.4%) followed by in-person and video chat visits combined (11.1%), all visits via video chat (5.6%), and all visits by phone only (2.8%).

### Caregiver and child weight

Caregivers’ median BMI decreased by 1.8% from baseline to post-assessment, a significant difference at the *p* < .05 level (Mdn 35.5, IQR 10.3, vs. Mdn 34.9, IQR 11.3, *p* = 0.016, *r* = 0.22). The majority (63.9%) of caregivers either maintained their weight (no change) or lost weight (reduction in kilograms from pre to post). Children’s BMI percentile showed a significant decrease from pre- to post-assessment (Mdn 98.8, IQR 3.5, vs. Mdn 98.7, IQR 3.4, *p* = 0.01, *r* = .20) as well as BMI percentile *z*-score (*p* = 0.023, *r* = .18). Children’s measured percent of the 95th percentile for sex and age at baseline (Mdn 118.3, IQR 35.8) and post-assessment (Mdn 121.9, IQR 31.8) remained stable (*p* = 0.050, *r* = 0.15); 65.1% of children either maintained or decreased their BMI percentile/BMIz while 41.0% of children either maintained or decreased in terms of percentage of the 95th percentile, highlighting the sensitivity of different calculations.

### Diet and walking

Caregivers and children both showed a significant decrease in median ounces of sugar-sweetened beverages consumed per day over the intervention period. Caregivers had a 43.7% decrease in median ounces per day (Mdn 10.1, IQR 27.1, vs. Mdn 5.7, IQR 14.6, *p* = 0.026, *r* = 0.22), and children decreased median ounces per day by 50.0% (Mdn 4.7, IQR 17.9, vs. Mdn 2.5, IQR 10.6, *p* = 0.006, *r* = 0.23).

Fruit and vegetable consumption remained stable from baseline to post-assessment. The median number of times caregivers consumed a fruit or vegetable in the previous day was 2.0 times (Q1–Q2, 2.0) at baseline and did not significantly differ at post-assessment (Mdn 2.0, IQR 2.0, *p* = 0.674, *r* = 0.04). Children also maintained similar fruit and vegetable consumption (Mdn 2.0, IQR 2.0, vs. Mdn 2.0, IQR 1.0, *p* = 0.388, *r* = 0.07).

Most caregivers reported walking two times or less in the past week for at least 10 min either to get to a place (84.8%) or for fun/exercise (70.2%) at baseline. Similar findings were present at post-assessment (80.0%, *p* = 0.724, and 75.9%, *p* = 0.752, *g* = 0.10, respectively). Likewise, children’s walking was not significantly different at post-assessment compared to baseline (walk to get some place 85.1% vs. 79.5%, *p* = 0.803, *g* = 0.06, and walk for fun/relaxation 75.8% vs. 73.4%, *p* = 0.480, *g* = 0.11).

### Home environment

The proportion of children who watched TV or used another computer screen for two or more hours per day significantly decreased from baseline to post-assessment (67.1 to 52.4%, *p* = 0.046, *g* = 0.22). Caregivers also showed a decrease in screen time at post-assessment, though not significantly different from baseline (60.7 to 52.5%, *p* = 0.332, *g* = 0.15).

The proportion of caregivers with soda in the home that is easy to access (either in plain sight or out of sight) reduced from baseline (60.7%) to post-assessment (40.0%), a significant decrease (*p* = 0.002, *g* = 0.43). Sweet and/or salty snacks also saw a reduction in proportion of caregivers reporting having them easy to get to (93.5 to 86.7%), though not significantly (*p* = 0.202, *g* = .33).

## Discussion

The purpose of this pilot study was to test the feasibility and efficacy of a tailored lifestyle intervention for caregivers and their child with obesity in partnership with Envolve, a national organization that reaches millions of Medicaid families. The results from this pilot study inform future interventions in several ways. First, this pilot demonstrated that a lifestyle change intervention, tailored to the context of Medicaid members and delivered by a peer coach through a minimal number of home visits, yielded a significant reduction in caregiver weight and reduction in child BMI percentile or maintenance of weight. Though reductions among children with BMI percentiles that are more than 100% of the 95th percentile may need more context. In addition, effect sizes were small for all pre-post changes in measures of BMI. Caregiver and child weight changes appeared to be achieved primarily due to reduction of sugar-sweetened beverage intake, supported by improvements in the food environment at home. The intervention did not impact other target behaviors such as fruit and vegetable consumption or increased walking. This is consistent with evidence that links higher intake of sugar-sweetened beverages to weight gain [59] and related chronic diseases such as type 2 diabetes [60, 61]. Weight is influenced by additional daily servings of sugar-sweetened beverages, with BMI increases by 0.06 kg m^−2^ and weight by 0.22 kg in children and adults, respectively [62]. These changes are particularly valuable since there is extensive evidence that caregivers influence the lifestyle behaviors of their child, in part through their control of the child’s food and activity environment [63], and this mirrors other research that suggests caregiver changes precede those of their child [64]. This supports an approach to simplify and focus content on select behaviors such as beverage intake, and promote home environment changes, which is needed to support and maintain significant change in this high-risk group.

In contrast, reductions in screen time by both caregiver and child did not translate to increases in walking bouts. There are several reasons why this might occur. Activity such as walking often involves not just home but neighborhood environments. Additionally, other work has suggested changes in activity among mothers take longer to modify than specific dietary changes [31]. Achieving success through simple dietary modifications, with ongoing support, might be needed to encourage the motivation for additional changes, followed by a focus on activity. More information is needed on how best to structure intervention content, and the value in a stepped versus combined approach in addressing lifestyle behaviors.

As importantly, this pilot suggests necessary insights on engagement of this Medicaid population in pediatric obesity interventions. Only 9% of the 1302 Medicaid families referred to this pilot program and who were ultimately able to be reached agreed to participate. Among those participating, 66% did not see barriers to addressing their child’s obesity despite limited economic resources; the remaining 34% were willing to participate but cited a range of barriers to their efforts (financial, food insecurity, housing). Common findings of low participation rates suggest more work is needed to reach caregivers, perhaps via channels through which they are already engaged, and to appropriately tailor interventions while considering the priorities of the family. This is consistent with other research completed with this population [9] and allows for an approximation of the generalizability of the pilot participants with other Medicaid recipients.

Caregiver engagement may also be influenced by the extent to which they believe obesity is a priority for their child [61]. Caregivers in this study had been informed by healthcare providers that their child had a diagnosis of obesity, the reason for the referral to Raising Well at Home. Yet 35% of caregivers did not agree that their child was obese. There are several possible explanations for this unexpected finding. The immediate social needs and competing priorities of this population may influence the ability to address obesity among children [9, 65]. This might also suggest caregivers hold a different perception of weight and body image, so did not agree with the information that their child had obesity. Perspectives regarding weight may also be influenced by sociocultural beliefs and lifestyle patterns that are ingrained over the life course of generations [7, 66]. Additionally, caregivers may not want to acknowledge that their child has obesity, which may be perceived as associated with caregiver actions or responsibility [67]. Further research is needed to better understand the barriers and facilitators of caregiver awareness and recognition of obesity in children and how it may influence support for lifestyle behavior change needed to promote healthy weight.

Finally, these data contribute to the literature showing the value of peer coaches in delivering interventions for under-resourced populations, particularly in the home setting [66]. Caregiver satisfaction with the intervention and the peer coach was overwhelmingly high, with the vast majority of participants recommending the program to others. Caregivers liked the limited number of home visits they received, and also maintained contact by texting with their peer coach. The face-to-face home visiting contact between the peer coach and caregiver and child impacted lifestyle behaviors and, importantly, changes in the home environment. Other work has shown that even a minimal number of home visits can yield improvements in lifestyle behaviors and weight [30] and that these improvements may be reinforced through home visits delivered over time. Texting and other technologies have also been recognized as a valuable strategy for supporting change [68]. This pilot provides further evidence of the feasibility and impact of a tailored lifestyle intervention delivered by peer coaches that can be delivered by peer coaches through Envolve, with the potential to reach caregivers and their children with obesity across the nation.

## Limitations

As this was a pilot, pre-post study, the lack of randomization and a comparison group may introduce bias in results. Generalizability may be reduced due to low recruitment and small sample size. Other limitations of this study include the use of self-report measures that may yield error in outcomes or biased results. Finally, the outcome analyses excluded participants that did not provide data after baseline, which might introduce bias in the results if the failure to provide post-baseline data was not completely at random.

## Conclusion

The intensity of behavioral interventions to achieve pediatric weight loss has shown limited feasibility in real-world settings or with under-resourced populations. Partnerships with organizations that serve under-resourced, Medicaid populations are critical to addressing the epidemic of childhood obesity. The Raising Well at Home pilot demonstrated that a tailored lifestyle interventions, delivered by peer coaches in the home and via text, are feasible and can improve weight, eating, and environmental measures of caregivers and children with obesity on Medicaid. Future work should determine the effectiveness, sustainability, and scalability of this intervention in sites located across the country.

## Data Availability

The datasets used and/or analyzed during the current study are available from the corresponding author on reasonable request.

## Abbreviations

BMI: Body mass index
CONSORT: Consolidation Standards of Reporting Trials

## References

[1] O’Connor EA, Evans CV, Burda BU, Walsh ES, Eder M, Lozano P (2017). Screening for obesity and interventions for weight management in children and adolescents.

[2] Kim K, Choi JS, Choi E, Nieman CL, Joo JH, Lin FR (2016). Effects of community-based health worker interventions to improve chronic disease management and care among vulnerable populations: a systematic review. American Journal of Public Health..

[3] Grossman DC, Bibbins-Domingo K, Curry SJ, Barry MJ, Davidson KW, Doubeni CA (2017). Screening for obesity in children and adolescents: US Preventive Services Task Force Recommendation Statement. JAMA.

[4] Simmonds M, Burch J, Llewellyn A, Griffiths C, Yang H, Owen C (2015). The use of measures of obesity in childhood for predicting obesity and the development of obesity-related diseases in adulthood: a systematic review and meta-analysis. Health Technol Assess.

[5] Tang TS, Ayala GX, Cherrington A, Rana G (2011). A review of volunteer-based peer support interventions in diabetes. Diabetes Spectrum..

[6] Krueger PM, Reither EN (2015). Mind the gap: race/ethnic and socioeconomic disparities in obesity. Current Diabetes Reports..

[7] Anderson SE, Keim SA (2016). Parent–child interaction, self-regulation, and obesity prevention in early childhood. Current Obesity Reports..

[8] Haire-Joshu D, Tabak R (2016). Preventing obesity across generations: evidence for early life intervention. Annual Review of Public Health..

[9] Kreuter MW, McQueen A, Boyum S, Fu Q (2016). Unmet basic needs and health intervention effectiveness in low-income populations. Preventive Medicine..

[10] Shilling V, Morris C, Thompson-Coon J, Ukoumunne O, Rogers M, Logan S (2013). Peer support for parents of children with chronic disabling conditions: a systematic review of quantitative and qualitative studies. Developmental Medicine & Child Neurology..

[11] Norris SL, Chowdhury FM, Van Le K, Horsley T, Brownstein JN, Zhang X (2006). Effectiveness of community health workers in the care of persons with diabetes. Diabet Med.

[12] Dutton GR, Lewis CE, Cherrington A, Pisu M, Richman J, Turner T (2018). A weight loss intervention delivered by peer coaches in primary care: rationale and study design of the PROMISE trial. Contemporary Clinical Trials..

[13] Palmas W, March D, Darakjy S, Findley SE, Teresi J, Carrasquillo O (2015). Community health worker interventions to improve glycemic control in people with diabetes: a systematic review and meta-analysis. Journal of General Internal Medicine..

[14] Viswanathan M, Kraschnewski JL, Nishikawa B, Morgan LC, Honeycutt AA, Thieda P, et al. Outcomes and costs of community health worker interventions: a systematic review. Med Care. 2010;48(9):792–808. 10.1097/MLR.0b013e3181e35b51 20706166

[15] Sullivan-Bolyai S, Bova C, Leung K, Trudeau A, Lee M, Gruppuso P (2010). Social support to empower parents (STEP). The Diabetes Educator..

[16] Postma J, Karr C, Kieckhefer G (2009). Community health workers and environmental interventions for children with asthma: a systematic review. Journal of Asthma..

[17] Schroeder K, McCormick R, Perez A, Lipman T (2018). The role and impact of community health workers in childhood obesity interventions: a systematic review and meta-analysis. Obes Rev.

[18] Leahey TM, Wing RR (2013). A randomized controlled pilot study testing three types of health coaches for obesity treatment: professional, peer, and mentor. Obesity.

[19] Katula JA, Vitolins MZ, Rosenberger EL, Blackwell CS, Morgan TM, Lawlor MS (2011). One-year results of a community-based translation of the Diabetes Prevention Program: Healthy-Living Partnerships to Prevent Diabetes (HELP PD) Project. Diabetes Care..

[20] West DS, Bursac Z, Cornell CE, Felix HC, Fausett JK, Krukowski RA (2011). Lay health educators translate a weight-loss intervention in senior centers: a randomized controlled trial. American Journal of Preventive Medicine..

[21] Tabak RG, Schwarz CD, Kemner A, Schechtman KB, Steger-May K, Byrth V (2019). Disseminating and implementing a lifestyle-based healthy weight program for mothers in a national organization: a study protocol for a cluster randomized trial. Implementation Science..

[22] Swindle T, Curran GM, Johnson SL (2019). Implementation science and nutrition education and behavior: opportunities for integration. J Nutr Educ Behav.

[23] McCrabb S, Lane C, Hall A, Milat A, Bauman A, Sutherland R, et al. Scaling-up evidence-based obesity interventions: a systematic review assessing intervention adaptations and effectiveness and quantifying the scale-up penalty. Obesity Reviews. 2019;20(7):964–82. 10.1111/obr.12845 30868745

[24] Hardy LL, Mihrshahi S, Gale J, Nguyen B, Baur LA, O’Hara BJ (2015). Translational research: are community-based child obesity treatment programs scalable?. BMC Public Health..

[25] Kumanyika S, Whitt-Glover M, Haire-Joshu D (2014). What works for obesity prevention and treatment in black Americans? Research directions. Obes Rev.

[26] Who We Are (2019). Centene Corporation.

[27] Centene, Corporation (2018). Annual Report. PDF.

[28] Home State Health Plan (2015). Raising Well Pediatric Obesity Program. Centene Corporation.

[29] Tabak RG, Dsouza N, Schwarz CD, Quinn K, Kristen P, Haire-Joshu D (2018). A formative study to understand perspectives of families eligible for a pediatric obesity program: a qualitative study. BMC Public Health..

[30] Haire-Joshu D, Elliott MB, Caito NM, Hessler K, Nanney M, Hale N (2008). High 5 for Kids: the impact of a home visiting program on fruit and vegetable intake of parents and their preschool children. Prev Med.

[31] Haire-Joshu D, Schwarz CD, Steger-May K, Lapka C, Schechtman K, Brownson RC (2018). A randomized trial of weight change in a national home visiting program. American Journal of Preventive Medicine..

[32] Haire-Joshu D, Cahill AG, Stein RI, Cade WT, Woolfolk CL, Moley K (2019). Randomized controlled trial of home-based lifestyle therapy on postpartum weight in underserved women with overweight or obesity. Obesity..

[33] Wing RR, Tate D, Espeland M, Gorin A, LaRose JG, Robichaud EF (2013). Weight gain prevention in young adults: design of the study of novel approaches to weight gain prevention (SNAP) randomized controlled trial. BMC Public Health.

[34] Glanz K, Rimer BK, Lewis FM (2002). Health behavior and health education: theory, research, and practice.

[35] Baranowski T, GS P., Glanz KLFRB (1997). How individuals, environments, and health behaviors interact. Health behavior and health education: theory, research, and practice.

[36] Sallis JF, FML KG, Rimer BK (1997). N O. Ecological models. Health behavior and health education: theory, research, and practice.

[37] Bell BA, Salkind NJ (2010). Pretest-posttest design. Encyclopedia of research design.

[38] Centers for Disease Control and Prevention (CDC) (2011). About BMI for adults.

[39] Centers for Disease Control and Prevention (CDC) (2011). About BMI for children and teens.

[40] Hedrick V, Myers E, Zoellner J, Duffey K, Davy B (2018). Validation of a rapid method to assess habitual beverage intake patterns. Nutrients..

[41] Hedrick VE, Comber DL, Estabrooks PA, Savla J, Davy BM (2010). The beverage intake questionnaire: determining initial validity and reliability. Journal of the American Dietetic Association..

[42] Hedrick VE, Comber DL, Ferguson KE, Estabrooks PA, Savla J, Dietrich AM (2013). A rapid beverage intake questionnaire can detect changes in beverage intake. Eating Behaviors..

[43] Hedrick VE, Savla J, Comber DL, Flack KD, Estabrooks PA, Nsiah-Kumi PA (2012). Development of a brief questionnaire to assess habitual beverage intake (BEVQ-15): sugar-sweetened beverages and total beverage energy intake. Journal of the Academy of Nutrition and Dietetics..

[44] Hill CE, MacDougall CR, Riebl SK, Savla J, Hedrick VE, Davy BM (2017). Evaluation of the relative validity and test–retest reliability of a 15-item beverage intake questionnaire in children and adolescents. J Acad Nutr Diet.

[45] O’Brien MJ, Davey A, Alos VA, Whitaker RC (2013). Diabetes-related behaviors in Latinas and non-Latinas in California. Diabet Care.

[46] Craig CL, Marshall AL, Sjöström M, Bauman AE, Booth ML, Ainsworth BE (2003). International physical activity questionnaire: 12-country reliability and validity. Med Sci Sports Exerc.

[47] Vaughn AE, Dearth-Wesley T, Tabak RG, Bryant M, Ward DS (2017). Development of a comprehensive assessment of food parenting practices: the home self-administered tool for environmental assessment of activity and diet family food practices Survey. J Acad Nutr Diet..

[48] Vaughn AE, Tabak RG, Bryant MJ, Ward DS (2013). Measuring parent food practices: a systematic review of existing measures and examination of instruments. Int J Behav Nutr Phys Act.

[49] Tabak RG, Morshed AB, Schwarz CD, Haire-Joshu D (2018). Impact of a healthy weight intervention embedded within a national home visiting program on the home food environment. Front Public Health.

[50] Kuczmarski RJ, Ogden CL, Guo SS, Grummer-Strawn LM, Flegal KM, Mei Z (2002). 2000 CDC Growth Charts for the United States: methods and development. Vital and health statistics Series 11, Data from the National Health Survey. Comparative Study.

[51] Centers for Disease Control and Prevention (CDC) (2014). A SAS Program for the 2000 CDC Growth Charts (ages 0 to<20 years).

[52] Field A (2013). Discovering statistics using IBM SPSS statistics: sage.

[53] Mangiafico S (2016). Summary and analysis of extension program evaluation in R.

[54] R Core Team (2019). R: a language and environment for statistical computing. Vienna, Austria: R Foundation for Statistical Computing.

[55] RStudio Team (2018). RStudio: integrated development for R.

[56] van Buuren S. Analysis of growth data. 0.39 ed. CRAN2018. p. Tools for the analysis of growth data: to extract an LMS table from a gamlss object, to calculate the standard deviation scores and its inverse, and to superpose two wormplots from different models. AGD: Analysis of Growth Data_. R package version 0.39. https://CRAN.R-project.org/package=AGD.

[57] Mangiafico S (2020). rcompanion: functions to support extension education program evaluation, version 2.3. 25.

[58] Vogel M. Data and methods around reference values in pediatrics. 0.7.4 ed. CRAN2019. p. Calculation of standard deviation scores and percentiles adduced from different growth standards (WHO, UK, Germany, Italy, China, etc). https://CRAN.Rproject.org/package=childsds.

[59] Te Morenga L, Mallard S, Mann J (2013). Dietary sugars and body weight: systematic review and meta-analyses of randomised controlled trials and cohort studies. BMJ.

[60] Malik VS, Popkin BM, Bray GA, Després J-P, Hu FB (2010). Sugar-sweetened beverages, obesity, type 2 diabetes mellitus, and cardiovascular disease risk. Circulation..

[61] Vargas-Garcia E, Evans C, Prestwich A, Sykes-Muskett B, Hooson J, Cade J (2017). Interventions to reduce consumption of sugar-sweetened beverages or increase water intake: evidence from a systematic review and meta-analysis. Obes Rev.

[62] Malik VS, Pan A, Willett WC, Hu FB (2013). Sugar-sweetened beverages and weight gain in children and adults: a systematic review and meta-analysis. The American Journal of Clinical Nutrition..

[63] Yee AZ, Lwin MO, Ho SS (2017). The influence of parental practices on child promotive and preventive food consumption behaviors: a systematic review and meta-analysis. Int J Behav Nutr Phys Act.

[64] Reicks M, Banna J, Cluskey M, Gunther C, Hongu N, Richards R (2015). Influence of parenting practices on eating behaviors of early adolescents during independent eating occasions: implications for obesity prevention. Nutrients..

[65] Braveman P, Gottlieb L (2014). The social determinants of health: it’s time to consider the causes of the causes. Public Health Rep.

[66] Appelhans B, Moss O, Cerwinske L (2016). Systematic review of paediatric weight management interventions delivered in the home setting. Obes Rev.

[67] Birch LL. Learning to eat: behavioral and psychological aspects. Preventive Aspects of Early Nutrition. 85. Basel: Karger Publishers; 2016. p. 125–34. 10.1159/000439503 27088340

[68] Kaakinen P, Kyngäs H, Kääriäinen M (2018). Technology-based counseling in the management of weight and lifestyles of obese or overweight children and adolescents: a descriptive systematic literature review. Inform Health Soc Care.

